# ﻿Telomere DNA in the insect order Dermaptera and the first evidence for the non-canonical telomeric motif TTCGG in Arthropoda

**DOI:** 10.3897/compcytogen.19.142613

**Published:** 2025-02-07

**Authors:** Vladimir A. Lukhtanov

**Affiliations:** 1 Department of Karyosystematics, Zoological Institute of the Russian Academy of Sciences, Universitetskaya nab. 1, St. Petersburg 199034, Russia Zoological Institute of the Russian Academy of Sciences St. Petersburg Russia

**Keywords:** Chromosomes, Dermaptera, genome assemblies, insects, telomere, telomeric motif, Zoraptera

## Abstract

Despite recent advances in telomere research, the telomere DNA organization remains unknown for representatives of several insect orders. In this study, analysis of the chromosome-level genome assembly shows that the telomeric DNA of the earwig *Labiaminor* (Linnaeus, 1758) (Polyneoptera, Dermaptera, Spongiphoridae) consists of repeats of the 5 bp motif TTCGG/CCGAA. This is the first record describing the structure of telomeric DNA in the order Dermaptera. This record expands the spectrum of the known telomeric sequences, since the TTCGG motif has not been reported for insects previously.

## ﻿Introduction

In many insects, the organization of telomeric DNA is highly conservative and, with a few exceptions, telomeric DNA is represented by a short TTAGG motif, which is repeated tens, hundreds, and even thousands of times at the ends of chromosomes. This telomeric motif is considered to be ancestral for the class Insecta and the entire phylum Arthropoda and is preserved in the majority of the studied insect orders and families (Frydrychová et al. 2004; Vítková et al. 2005; Lukhtanov and Kuznetsova 2010; Kuznetsova et al. 2020).

Recent studies have confirmed the widespread and frequent occurrence of the TTAGG motif (Gokhman and Kuznetsova 2018; Grozeva et al. 2019; Prušáková et al. 2021; Lukhtanov and Pazhenkova 2023; Bugrov et al. 2024). At the same time, they have shown that insects are characterized by a much greater diversity in the organization of telomeric DNA than was previously assumed. It has been shown that in addition to the canonical TTAGG motif, insects have a large number of other variants of telomeric repeats, the length of which varies from 1 to 11 nucleotides (Lukhtanov 2022; Zhou et al. 2022; Fajkus et al. 2023; Lukhtanov and Pazhenkova 2023; Lyčka et al. 2024; Stoianova et al. 2024). In some representatives of the Diptera families Syrphidae and Tachinidae, longer (173–381 bp) repeats are found at chromosome ends (Lukhtanov and Pazhenkova 2023). It has been also established that in most insects the arrays of the short telomeric motifs are interspersed with telomere-specific non-LTR retrotransposons of the SART and TRAS families (Kubo et al. 2001; Fujiwara et al. 2005; Osanai et al. 2006; Kirkness et al. 2010; Monti et al. 2013; Lukhtanov and Pazhenkova 2023; Pazhenkova and Lukhtanov 2023).

Despite the above-mentioned advances in the study of telomeric DNA in insects, the structure of telomeres remains unknown for representatives of many orders. This complicates the reconstruction of the ancestral organization of insect chromosomes and the study of the evolution of mechanisms that maintain telomere length. In particular, there are still no data on the organization of telomeric DNA in earwigs (the order Dermaptera) (Kuznetsova et al. 2020). This order is especially intriguing from the point of view of telomere organization. An attempt has been made to elucidate the structure of telomeric motif in the earwig *Forficulaauricularia* Linnaeus, 1758 using the fluorescence *in situ* hybridization (FISH) method and the TTAGG probe (Frydrychová et al. 2004); however, it was unsuccessful. Two attempts to elucidate the telomere structure in earwigs using Southern blot hybridization (Okazaki et al. 1993; Frydrychová et al. 2004) were also unsuccessful. These attempts can be interpreted as evidence that the TTAGG motif is absent from the telomeres of the studied species. At the same time, they did not bring us any closer to understanding how telomeres are organized in earwigs.

In this study an analysis of the chromosome-level genome assembly of *Labiaminor* (Linnaeus, 1758) (Spongiphoridae) and scaffold-level genome assemblies of *Forficulaauricularia* (Forficulidae), *Euborelliaannulipes* (H. Lucas, 1847) (Anisolabididae), and *Anisolabismaritima* (Bonelli, 1832) (Anisolabididae) was carried out to enable investigation of the telomeric DNA of Dermaptera.

## ﻿Material and methods

Chromosome-level genome assembly of *Labiaminor* generated by the Darwin Tree of Life Project (https://www.darwintreeoflife.org/) (2022) and freely available upon deposition in the European Nucleotide Archive (ENA) (https://www.darwintreeoflife.org/wp-content/uploads/2020/03/DToL-Open-Data-Release-Policy-1.pdf) was used for search and analysis of telomere and subtelomere sequences. Nucleotide sequences representing each of the seven chromosomes of this species were downloaded from GenBank (Table 1). Their terminal regions were visually inspected.

In addition, scaffold-level genome assemblies *Forficulaauricularia*, *Euborelliaannulipes*, and *Anisolabismaritima* were examined. Telomere-like sequences in these genomes were searched using the GenBank *blastn* function with the “Low complexity region” filter disabled and the (TTAGG)_30_ and (TTCGG)_30_ sequences as queries.

## ﻿Results and discussion

In the analyzed assembly, I found no evidence of telomeric motifs in chromosomes 1, 2, 4, 6 and in the left ends of chromosomes 5 and X (Table 1). This situation is not unexpected. Telomeric sequences at chromosome ends are the most difficult genome regions to assemble with accuracy and completeness (Rhie et al. 2021; Kim et al. 2022). Therefore, some assemblies may be truncated at the ends of chromosomes or even contain no telomeric repeats at all. Even if telomeric repeats are present, it is difficult to be sure that these sequences are complete and contain the correct number of copies.

**Table 1.** The structure of the terminal ends in chromosomes of *L.minor*.

Chromosome	Left telomere	Right telomere	Chromosome size (bp)	GenBank
1	truncated	truncated	126 263 735	OY720344.1
2	truncated	truncated	95 403 478	OY720345.1
3	**CCGAA**	**TTCGG**	86 446 126	OY720346.1
4	truncated	truncated	83 479 239	OY720347.1
5	truncated	**TTCGG**	70 759 738	OY720349.1
6	truncated	truncated	60 004 694	OY720350.1
X	truncated	**TTCGG**	76 888 567	OY720348.1

However, if telomeric motifs are conserved on some chromosomes or at the ends of some chromosomes, they can be easily recognized by a combination of the following features. (1) Each telomeric end (if conserved in whole or in part) is represented by a large number of exactly identical short repeats. (2) These end structures, if present in the assembly, consist of the same motifs on different chromosomes. (3) If telomeric motifs are present at the left and right ends of the chromosome, they have a reverse-complement structure and orientation. (4) Short telomere-like motifs are rare in interstitial positions of chromosomes, and if they occur there, then in a small number of copies.

In the case of the *L.minor* chromosome assembly, the TTCGG telomeric motif was found in the most terminal positions of the right ends of chromosomes 3, 5 and X. In addition, the CCGAA motif, which is a reverse-complement sequence to TTCGG, was found in the terminal left end of chromosome 3 (Table 1). Each of these terminal sequences was represented by a large number of TTCGG/CCGAA repeats (from 351 in chromosome 5 to 481 in the left end of chromosome 3). In interstitial positions of all chromosomes, even short (TTCGG/CCGAA)_3_ and (TTCGG/ CCGAA)_4_ repeats were rare. Based on this, I interpret the long sequences of short TTCGG/CCGAA motifs at the ends of chromosomes 3, 5, and X as telomeric sequences. The probability that these sequences represent a random combination of non-telomeric nucleotides seems vanishingly small.

In addition, I studied the subtelomeric regions of chromosomes bordering telomeric sequences. This analysis showed that the subtelomeric regions are rich in variant repeats of TTCAGTT and TTCGGTT. The presence of short repeats that are close in structure to telomeric motifs, but are slightly altered, was previously noted as one of the characteristic features of subtelomeric regions (Louis and Vershinin 2005). For the species *Forficulaauricularia*, *Euborelliaannulipes*, and *Anisolabismaritima*, in the GenBank, there are only scaffold-level genome assemblies that include no assembled chromosomes. A blast of these assemblies using the (TTCGG)_30_ sequence as a query revealed that these assemblies contained extended arrays of (TTCGG)_n_. Blast of these assemblies using the (TTAGG)_30_ sequence did not reveal extended arrays of (TTAGG)_n_. This result suggests that the TTCGG telomeric motif is most likely present in these species and is therefore widespread in the order Dermaptera.

The obtained result seems interesting in several respects. It is the first result showing the structure of telomeric DNA in the order Dermaptera. In addition, it expands the spectrum of the known telomeric sequences, since the TTCGG motif has not been reported for insects previously. Thirdly, this motif may be a synapomorphy marking either all the studied families (Spongiphoridae, Forficulidae and Anisolabididae), or all earwigs, or even a phylogenetic lineage of a higher rank. Earwigs belong to a monophyletic group of orders called Polyneoptera. For other orders of this group, the presence of the canonical telomeric motif TTAGG has been shown (Kuznetsova et al. 2020). The presence of the TTCGG motif may be a feature indicating an isolated position of earwigs. The order Dermaptera is sister to the enigmatic and poorly studied order Zorapera (Wipfler et al. 2019). Therefore, it would be interesting to elucidate the telomere organization in Zoraptera in the future.

## References

[B1] Bugrov A Karamysheva T Buleu O (2024) New insights into the chromosomes of stoneflies: I. Karyotype, C-banding and localization of ribosomal and telomeric DNA markers in *Skwalacompacta* (McLachlan, 1872) (Polyneoptera, Plecoptera, Perlodidae) from Siberia. Comparative Cytogenetics 18: 15–26. 10.3897/compcytogen.18.115784 38313463 PMC10835800

[B2] Fajkus P Adámik M Nelson ADL Kilar AM Franek M Bubeník M Frydrychová RČ Votavová A Sýkorová E Fajkus J Peška V (2023) Telomerase RNA in Hymenoptera (Insecta) switched to plant/ciliate-like biogenesis. Nucleic Acids Research 51: 420–433. https://doi.org.10.1093/nar/gkac1202 36546771 10.1093/nar/gkac1202 PMC9841428

[B3] Frydrychová R Grossmann P Trubač P Vítková M Marec F (2004) Phylogenetic distribution of TTAGG telomeric repeats in insects. Genome 47: 163–178. https://doi.org.10.1139/g03-100 15060613 10.1139/g03-100

[B4] Fujiwara H Osanai M Matsumoto T Kojima KK (2005) Telomere-specific non-LTR retrotransposons and telomere maintenance in the silkworm, *Bombyxmori*. Chromosome Research 13: 455–467. 10.1007/s10577-005-0990-9 16132811

[B5] Gokhman VE Kuznetsova VG (2018) Presence of the canonical TTAGG insect telomeric repeat in the Tenthredinidae (Symphyta) suggests its ancestral nature in the order Hymenoptera. Genetica 146: 341–344. 10.1007/s10709-018-0019-x 29730744

[B6] Grozeva S Anokhin BA Simov N Kuznetsova VG (2019) New evidence for the presence of the telomeric motif (TTAGG)n in the family Reduviidae and its absence in the families Nabidae and Miridae (Hemiptera, Cimicomorpha). Comparative Cytogenetics 13: 283–295. https://doi.org.10.3897/CompCytogen.v13i3.36676 31579434 10.3897/CompCytogen.v13i3.36676 PMC6765027

[B7] Kim J Lee C Ko BJ Yoo DA Won S Phillippy AM Fedrigo O Zhang G Howe K Wood J Durbin R Formenti G Brown S Cantin L Mello CV Cho S Rhie A Kim H Jarvis ED (2022) False gene and chromosome losses in genome assemblies caused by GC content variation and repeats. Genome Biology 2: 204. 10.1186/s13059-022-02765-0 PMC9516821 36167554

[B8] Kirkness EF Haas BJ Sun W Braig HR Perotti MA Clark JM Lee SH Robertson HM Kennedy RC Elhaik E Gerlach D Kriventseva EV Elsik CG Graur D Hill CA Veenstra JA Walenz B Tubío JM Ribeiro JM Rozas J Johnston JS Reese JT Popadic A Tojo M Raoult D Reed DL Tomoyasu Y Kraus E Mittapalli O Margam VM Li HM Meyer JM Johnson RM Romero-Severson J Vanzee JP Alvarez-Ponce D Vieira FG Aguadé M Guirao-Rico S Anzola JM Yoon KS Strycharz JP Unger MF Christley S Lobo NF Seufferheld MJ Wang N Dasch GA Struchiner CJ Madey G Hannick LI Bidwell S Joardar V Caler E Shao R Barker SC Cameron S Bruggner RV Regier A Johnson J Viswanathan L Utterback TR Sutton GG Lawson D Waterhouse RM Venter JC Strausberg RL Berenbaum MR Collins FH Zdobnov EM Pittendrigh BR (2010) Genome sequences of the human body louse and its primary endosymbiont provide insights into the permanent parasitic lifestyle. Proceedings of the National Academy of Sciences of the United States of America 107: 12168–12173. 10.1073/pnas.1003379107 20566863 PMC2901460

[B9] Kubo Y Okazaki S Anzai T Fujiwara H (2001) Structural and phylogenetic analysis of TRAS, telomeric repeat-specific non-LTR retrotransposon families in Lepidopteran insects. Molecular Biology and Evolution 18: 848–857. 10.1093/oxfordjournals.molbev.a003866 11319268

[B10] Kuznetsova V Grozeva S Gokhman V (2020) Telomere structure in insects: a review. Journal of Zoological Systematics and Evolutionary Research 58: 127–158. 10.1111/jzs.12332

[B11] Louis EJ Vershinin AV (2005) Chromosome ends: different sequences may provide conserved functions. Bioessays 27: 685–697. 10.1002/bies.20259 15954099

[B12] Lukhtanov VA Pazhenkova EA (2023) Diversity and evolution of telomeric motifs and telomere DNA organization in insects. Biological Journal of the Linnean Society 140: 536–555. https://doi.org.10.1093/biolinnean/blad068

[B13] Lukhtanov VA Kuznetsova VG (2010) What genes and chromosomes say about the origin and evolution of insects and other arthropods? Russian Journal of Genetics 46: 1115–1121. 10.1134/S1022795410090279 21061630

[B14] Lukhtanov VA (2022) Diversity and evolution of telomere and subtelomere DNA sequences in insects. bioRxiv 10.1101/2022.04.08.487650

[B15] Lyčka M Bubeník M Závodník M Peska V Fajkus P Demko M Fajkus J Fojtová M (2024) TeloBase: a community-curated database of telomere sequences across the tree of life. Nucleic Acids Research 52(D1): D311-D321. https://doi.org.10.1093/nar/gkad672 10.1093/nar/gkad672 PMC10767889 37602392

[B16] Monti V Serafini C Manicardi GC Mandrioli M (2013) Characterization of non-LTR retrotransposable TRAS elements in the aphids *Acyrthosiphonpisum* and *Myzuspersicae* (Aphididae, Hemiptera). Journal of Heredity 104: 547–553. 10.1093/jhered/est017 23530141

[B17] Okazaki S Tsuchida K Maekawa H Ishikawa H Fujiwara H (1993) Identification of a pentanucleotide telomeric sequence, (TTAGG)_n_, in the silkworm *Bombyxmori* and in other insects. Molecular and Cellular Biology 13: 1424–1432. 10.1128/MCB.13.3.1424 8441388 PMC359452

[B18] Osanai M Kojima KK Futahashi R Yaguchi S Fujiwara H (2006) Identification and characterization of the telomerase reverse transcriptase of *Bombyxmori* (silkworm) and *Triboliumcastaneum* (flour beetle). Gene 376: 281–289. 10.1016/j.gene.2006.04.022 16793225

[B19] Pazhenkova EA Lukhtanov VA (2023) Chromosomal conservatism vs chromosomal megaevolution: enigma of karyotypic evolution in Lepidoptera. Chromosome Research 31: 16. https://doi.org.10.1007/s10577-023-09725-9 10.1007/s10577-023-09725-9 37300756

[B20] Prušáková D Peska V Pekár S Bubeník M Čížek L Bezděk A Čapková Frydrychová R (2011) Telomeric DNA sequences in beetle taxa vary with species richness. Scientific Reports 11: 1331. 10.1038/s41598-021-92705-y PMC8233369 34172809

[B21] Rhie A McCarthy SA (2021) Towards complete and error-free genome assemblies of all vertebrate species. Nature 592: 737–746. 10.1038/s41586-021-03451-0 33911273 PMC8081667

[B22] Stoianova D Grozeva S Golub NV Anokhin BA Kuznetsova VG (2024) The first FISH confirmed non-canonical telomeric motif in Heteroptera: *Cimexlectularius* Linnaeus, 1758 and *C.hemipterus* (Fabricius, 1803) (Hemiptera, Cimicidae) have a 10 bp motif (TTAGGGATGG)_n_. Genes 15: 1026. 10.3390/genes15081026 PMC11354137 39202386

[B23] The Darwin Tree of Life Project Consortium (2022) Sequence locally, think globally: The Darwin tree of life project. Proceedings of the National Academy of Sciences of the United States of America 119: e2115642118. 10.1073/pnas.2115642118 PMC8797607 35042805

[B24] Vítková M Král J Traut W Zrzavý J Marec F (2005) The evolutionary origin of insect telomeric repeats, (TTAGG)_n_. Chromosome Research 13: 145–156. 10.1007/s10577-005-7721-0 15861304

[B25] Wipfler B Letsch H (2019) Evolutionary history of Polyneoptera and its implications for our understanding of early winged insects. Proceedings of the National Academy of Sciences of the United States of America 116: 3024–3029. https://doi.org.10.1073/pnas.1817794116 30642969 10.1073/pnas.1817794116 PMC6386694

[B26] Zhou Y Wang Y Xiong X Appel AG Zhang C Wang X (2022) Profiles of telomeric repeats in Insecta reveal diverse forms of telomeric motifs in Hymenopterans. Life Science Alliance 5: e202101163. 10.26508/lsa.202101163 PMC8977481 35365574

